# Distillers’ Grains-Derived Bioactive Fraction Suppresses Colorectal Cancer Progression Through Modulation of Autophagy-Associated PI3K/AKT Signaling

**DOI:** 10.3390/foods15162834

**Published:** 2026-08-14

**Authors:** Ning An, Qian Liu, Jinmiao Tian, Xiaxia Fan, Hanqing Li, Shuhua Shan, Jiangying Shi, Zhuoyu Li

**Affiliations:** 1 Institute of Biotechnology, Key Laboratory of Chemical Biology and Molecular Engineering of National Ministry of Education, Shanxi University, Taiyuan 030006, China; a3063789495@163.com (N.A.); tjm8235003@163.com (J.T.); fanxiaxia@sxu.edu.cn (X.F.); ssh@sxu.edu.cn (S.S.); shijy@sxu.edu.cn (J.S.); 2 School of Life Science, Shanxi University, Taiyuan 030006, China; liuq127@163.com (Q.L.); 13903461328@139.com (H.L.)

**Keywords:** bioactive fraction, colorectal cancer, autophagy, PI3K/AKT

## Abstract

Fenjiu distillers’ grains are a major by-product of Fenjiu production and contain various bioactive components derived from cereal raw materials and microbial fermentation. Colorectal cancer (CRC) remains a global health challenge, and the development of safe and effective therapeutic agents is urgently needed. In this study, we successfully obtained DGAE-1 (distillers’ grains ethanol extract fraction 1), an ethanol-extracted bioactive fraction derived from Fenjiu distillers’ grains, with anti-colorectal cancer activity. LC-MS analysis tentatively annotated the chemical constituents of DGAE-1 fraction based on MS/MS fragmentation patterns and database similarity matching, with the major annotated compounds belonging to carboxylic acids and derivatives, organic nitrogen compounds, organic phosphoric acid derivatives, and other chemical classes. These annotated compounds may contribute to the biological activity of DGAE-1 fraction, although the specific active constituents remain to be further clarified. DGAE-1 fraction inhibited colorectal cancer cell proliferation and induced autophagy. Further studies indicated that the PI3K/AKT pathway is involved in DGAE-1 fraction-induced autophagy. The results of this study demonstrate that DGAE-1 fraction exerts anti-colorectal cancer activity in both cellular and animal models. These findings provide new insights into the development and utilization of Fenjiu distillers’ grains and highlight their potential as a source of bioactive compounds for health-related applications.

## 1. Introduction

Fenjiu is a traditional Chinese baijiu produced in Fenyang, Shanxi Province, and is widely recognized as a representative of the light-aroma style baijiu [1]. Fenjiu distillers’ grains are the major by-products generated during the fermentation and distillation of cereal raw materials in Fenjiu production [2]. Unlike strong-aroma baijiu, which uses mud pit fermentation [3], or sauce-aroma baijiu, which employs high-temperature pile fermentation [4], Fenjiu utilizes solid-state fermentation in earthen jars, creating a relatively isolated fermentation environment that fosters a distinctive microbial community structure [5]. Studies have shown that during the Fenjiu fermentation process, functional microorganisms such as lactic acid bacteria, Bacillus, Weissella, and yeast are predominantly enriched [5,6]. These microbes secrete extracellular enzymes like amylase and protease, promoting the degradation and transformation of large-molecule substances in the raw materials [7]. A significant amount of metabolic byproducts—including free amino acids, small peptides, organic acids, and phenolic compounds—generated during fermentation remain partially retained in the distillers’ grain [8]. Therefore, Fenjiu distillers’ grains not only contain abundant grain-derived nutrients but also accumulate various bioactive substances formed through microbial metabolism, offering substantial potential for utilization and development [9].

Despite this potential, Fenjiu distillers’ grains are still largely underutilized, with most being used as animal feed or fertilizer [2]. In recent years, several studies have reported bioactivities, including antioxidant and gut-modulatory effects, from crude extracts [10,11,12]. Collectively, these findings suggest that distillers’ grains may serve as a promising source of bioactive substances for disease prevention and intervention. However, the chemical identities of the bioactive constituents remain unclear, and the analytical methods used have not been fully disclosed. This makes it difficult to attribute the observed effects to specific molecules or to ensure reproducibility. Therefore, systematic chemical characterization of distillers’ grains-derived active fractions and the establishment of reliable chemical profiles are essential prerequisites for further biological evaluation.

Colorectal cancer (CRC) is one of the most prevalent and deadly malignancies worldwide [13]. Although considerable progress has been made in the treatment of CRC, drug resistance, adverse effects, and tumor recurrence remain major clinical challenges [14]. Autophagy is a highly conserved intracellular degradation and recycling process that plays a complex dual role in tumor development and progression [15]. On the one hand, autophagy enables tumor cells to adapt to unfavorable conditions such as nutrient deprivation and hypoxia. On the other hand, excessive activation of autophagy can promote tumor cell death and thereby exert antitumor effects. Consequently, induction of cytotoxic autophagy has emerged as a promising strategy for cancer therapy [16]. In recent years, an increasing number of naturally derived bioactive compounds have been reported to suppress tumor growth through the regulation of autophagy [17]. Previous studies have shown that distillers’ grains-derived extracts exhibit anti-cancer activity, which has been associated with apoptotic cell death [18]. However, whether distillers’ grains-derived bioactive fractions can inhibit colorectal cancer progression through autophagy regulation has not been fully explored. Therefore, identifying bioactive fractions from distillers’ grains that can regulate autophagy may provide new insights into the high-value utilization of this brewing by-product and expand the potential sources of naturally derived anti-colorectal cancer agents. The PI3K/AKT signaling pathway is frequently dysregulated in colorectal cancer and is closely associated with autophagy regulation [19], suggesting that this pathway may be involved in the biological effects of bioactive fractions derived from distillers’ grains.

The aim of this study was to obtain an active fraction (DGAE-1) from Fenjiu distillers’ grains and investigate its anti-colorectal cancer activity and potential molecular mechanism. This study provides new insights into the utilization of distillers’ grains-derived bioactive fractions and supports their further exploration as potential resources for colorectal cancer intervention.

## 2. Materials and Methods

### 2.1. Reagent Material

The experimental distiller’s grains were taken from Shanxi Xinghuacun Fen Distillery Co., Ltd. (Fenyang, China). Human normal colon epithelial cell line FHC, human colorectal adenocarcinoma cell line SW620 and human colorectal adenocarcinoma cancer cell line HT-29 were purchased from the Cell Bank of the Chinese Academy of Sciences. Cell culture consumables, including cell culture flasks and dishes, were purchased from Guangzhou Jet Bio-Filtration Co., Ltd. (Guangzhou, China). RPMI-1640 medium and DMEM/F12 (1:1) medium were purchased from Procell Life Science & Technology Co., Ltd. (Wuhan, China). Fetal bovine serum was purchased from Gibco (Thermo Fisher Scientific, Grand Island, NY, USA). Penicillin-streptomycin-gentamycin mixed solution and 3-(4,5-Dimethylthiazol-2-yl)-2,5-diphenyltetrazolium bromide (MTT) were purchased from Beijing Solarbio Science & Technology Co., Ltd. (Beijing, China). Chloroquine (CQ) was purchased from Sigma-Aldrich (Merck, Darmstadt, Germany). 740Y-P was purchased from MedChemExpress (Monmouth Junction, NJ, USA). mCherry-LC3B plasmid was constructed by our research group in the previous stage.

### 2.2. Preparation of Dgae-1 Fraction

The distillers’ grains powder was extracted with 60% ethanol aqueous solution at a solid-to-liquid ratio of 1:25 using ultrasonic-assisted extraction (300 W) for 30 min. The extract was centrifuged at 11,000 rpm for 10 min at 4 °C, and the supernatant was collected and stored at 4 °C. The precipitate was re-extracted under the same conditions. The supernatants from the two extraction cycles were combined and concentrated by rotary evaporation at 65 °C to remove ethanol, yielding the distillers’ grains ethanol extract (DGAE). The extract was further filtered through a 0.22 μm membrane filter to remove impurities and stored at −20 °C until further use. The obtained DGAE was further purified using macroporous adsorption resins. DGAE was loaded onto weakly polar AB-8 macroporous adsorption resin (Mitsubishi Chemical Corporation, Tokyo, Japan) at a loading capacity of less than 50 mg/g resin. The resin was first washed with 3 bed volumes (BV) of ultrapure water at a flow rate of 1–2 BV/h to collect the flow-through fraction. Subsequently, ethanol solutions (30%, 60%, and 90%, *v*/*v*) were used for gradient elution, and the corresponding eluted fractions were collected. After removal of the solvent by rotary evaporation at 65 °C, the fractions were evaluated for anti-colorectal cancer activity. Based on the activity results, the AB-8 flow-through fraction was further purified using nonpolar SP207 macroporous adsorption resin (Mitsubishi Chemical Corporation, Japan). The SP207 resin was treated using the same procedure, and the flow-through fraction was collected after ultrapure water washing, followed by elution with 90% ethanol. The obtained active fraction was concentrated, lyophilized, and stored at −20 °C until further use, and was designated as DGAE-1. Three independent batches of DGAE-1 fraction were prepared using the same procedure, and their anti-colorectal cancer activity was evaluated using the MTT assay to confirm the consistency of biological activity among batches. The preparation process of the DGAE-1 fraction from Fenjiu distillers’ grains is illustrated in Figure S1.

### 2.3. LC-MS Analysis

The chemical composition of DGAE-1 fraction was analyzed using liquid chromatography–mass spectrometry (LC-MS). For sample preparation, 100 mg of thoroughly mixed DGAE-1 powder was transferred into a 2 mL centrifuge tube. Subsequently, 1 mL of 70% methanol and 3-mm steel beads were added, and the mixture was homogenized using a fully automatic rapid sample grinder (JXFSTPRP-48, 70 Hz, Shanghai Jingxin Industrial Development Co., Ltd., Shanghai, China) for 3 min. After cooling, the samples were subjected to low-temperature ultrasonication (40 kHz) for 10 min. The extracts were centrifuged at 12,000 rpm for 10 min at 4 °C, and the supernatants were collected and diluted 2–100-fold. Then, 10 μL of internal standard solution (100 μg/mL) was added, and the samples were filtered through a 0.22 μm PTFE membrane before LC-MS analysis. LC-MS analysis was performed using a Thermo Vanquish UHPLC system coupled with a Q Exactive HF high-resolution mass spectrometer (Thermo Fisher Scientific, Waltham, MA, USA). Chromatographic separation was achieved using a Zorbax Eclipse C18 column (1.8 μm × 2.1 mm × 100 mm, Agilent Technologies, Santa Clara, CA, USA) maintained at 30 °C. The mobile phase consisted of water containing 0.1% formic acid (A) and acetonitrile (B). The flow rate was set at 0.3 mL/min, with an injection volume of 2 μL, and the autosampler temperature was maintained at 4 °C. The gradient elution program was as follows: 5% B (0–2 min), 5–30% B (2–6 min), 30% B (6–7 min), 30–78% B (7–12 min), 78% B (12–14 min), 78–95% B (14–17 min), 95% B (17–20 min), 95–5% B (20–21 min), and 5% B (21–25 min). Mass spectrometry analysis was conducted using electrospray ionization (ESI) in both positive and negative ion modes. The ion source parameters were set as follows: heater temperature, 325 °C; sheath gas flow, 45 arb; auxiliary gas flow, 15 arb; sweep gas flow, 1 arb; spray voltage, 3.5 kV; capillary temperature, 330 °C; and S-Lens RF level, 55%. Full-scan MS data were acquired over an m/z range of 100–1500 with resolutions of 120,000 for MS1 and 60,000 for MS2. Data-dependent MS/MS acquisition (dd-MS^2^, Top N = 10) was performed using higher-energy collisional dissociation (HCD). The raw LC-MS data were processed using Compound Discoverer 3.3 for peak extraction, retention time correction, and molecular feature annotation. Metabolite annotation was performed based on accurate precursor mass, retention time, and MS/MS fragmentation patterns, followed by spectral matching against the Thermo mzCloud online database and mzVault local database. Since authentic standards were unavailable for most detected compounds, the annotated metabolites were considered tentative annotations rather than confirmed identifications. The relative abundance of each annotated metabolite was calculated based on the ratio of its peak area to the total peak area of all annotated metabolites. The relative percentages were used to describe the relative distribution of chemical components in DGAE-1 fraction and did not represent the absolute contents of individual compounds. The total ion chromatogram (TIC) of the DGAE-1 fraction obtained under positive electrospray ionization mode (ESI+) is shown in Figure S2. The compounds tentatively annotated based on LC-MS analysis are summarized in Table 1.

### 2.4. Cell Culture and MTT Assay

HT-29 and SW620 cells were maintained in RPMI-1640 medium, while FHC cells were cultured in DMEM/F12 medium. Both media were supplemented with 10% FBS and 1% penicillin–streptomycin- gentamycin. Cells were cultured at 37 °C in a 5% CO_2_ incubator. Cells in the logarithmic growth phase at approximately 90% confluence were trypsinized and seeded into 96-well plates at a density of 5 × 10^3^ cells per well. After cell attachment for 24 h, cells were treated with different concentrations of DGAE, DGAE-1 fraction, and other fractions obtained from the extraction and purification process, with five replicate wells for each concentration. After 48 h of treatment, 15 μL of MTT solution was added to each well and incubated for an additional 4 h. The culture medium was then removed, and 150 μL of DMSO was added to each well to dissolve the formazan crystals. The plates were gently shaken for 10 min, and the absorbance was measured at 570 nm using a microplate reader. The average absorbance value of the five replicate wells was used to calculate cell viability. For autophagy inhibition and PI3K/AKT activation experiments, cells were pretreated with chloroquine (CQ, 10 μM) or 740Y-P (10 μg/mL) for 1 h, followed by treatment with DGAE-1 fraction. After 48 h of co-treatment, cell viability was evaluated using the MTT assay as described above.

### 2.5. Colony Formation Assay

HT-29 and SW620 cells were plated into 24-well plates at a density of 5 × 10^3^ cells per well. After attachment for 24 h, the cells were treated with different concentrations of DGAE or DGAE -1 fraction for 5–7 days, with at least four replicate wells for each group. No medium replacement was performed during the treatment period. At the end of treatment, the medium was removed and the cells were fixed with 4% paraformaldehyde for 30 min, followed by staining with 0.1% crystal violet for 15 min. The plates were air-dried, and images were captured using an SZX16 stereomicroscope (Olympus, Tokyo, Japan). Colony formation was quantified by measuring the stained area with ImageJ 1.54 software. The experiment was independently repeated at least three times, and quantified data were expressed as mean ± SD and subjected to statistical analysis. For CQ and 740Y-P treatment experiments, cells were treated with CQ (10 μM) or 740Y-P (10 μg/mL), followed by DGAE-1 fraction treatment, and colony formation assays were performed as described above.

### 2.6. Apoptosis Analysis by Flow Cytometry

HT-29 and SW620 cells were seeded in 6-well plates, allowed to adhere for 24 h, and treated with different concentrations of DGAE or DGAE -1 fraction for 48 h. Both floating and adherent cells were collected, washed twice with cold PBS, and resuspended in 195 μL of binding buffer. Cells were stained with 5 μL of Annexin V-FITC and 5 μL of PI using an Annexin V-FITC/PI Apoptosis Detection Kit (Beyotime Biotechnology, Shanghai, China) and incubated for 15 min at room temperature in the dark. Samples were analyzed using a NovoCyte flow cytometer (Agilent Technologies, Santa Clara, CA, USA), with 10,000 events acquired per sample. Compensation was established using single-stained controls. Cell debris and aggregates were excluded based on FSC/SSC gating, and viable, early apoptotic, late apoptotic, and necrotic cells were defined as Annexin V−/PI−, Annexin V+/PI−, Annexin V+/PI+, and Annexin V−/PI+, respectively.

### 2.7. Western Blot Assay

Cells were lysed and total proteins were extracted. Protein concentrations were determined using a BCA protein assay kit (Beyotime Biotechnology, Nanjing, China). Equal amounts of protein samples were separated by SDS-PAGE and transferred onto PVDF membranes. After blocking with 5% skim milk, the membranes were incubated with the corresponding primary antibodies overnight at 4 °C. After washing, the membranes were incubated with HRP-conjugated secondary antibodies for 2 h at room temperature. Protein bands were visualized using an enhanced chemiluminescence (ECL) detection system, and band intensities were quantified using ImageJ 1.54 software. The expression levels of target proteins were normalized to β-actin or GAPDH, while phosphorylated proteins were normalized to their corresponding total protein levels.

The primary antibodies used for Western blot analysis included antibodies against LC3B (18725-1-AP), p62/SQSTM1 (18420-1-AP), PI3K (21866-1-AP), phosphorylated PI3K (p-PI3K, Tyr458), AKT (10176-2-AP), phosphorylated AKT (p-AKT, Ser473), mTOR (20657-1-AP), phosphorylated mTOR (p-mTOR, Ser2448), ULK1 (20986-1-AP), phosphorylated ULK1 (p-ULK1, Ser757), Beclin-1 (11306-1-AP), ATG5 (10181-2-AP), cleaved caspase-3 (19677-1-AP), cleaved caspase-8 (85171-3-RR), cleaved caspase-9 (23821-1-AP), β-actin (66009-1-Ig), and GAPDH (10494-1-AP) (Wuhan Sanying Biotechnology Co., Ltd. Wuhan, China). All primary antibodies were used at a dilution of 1:1000. HRP-conjugated secondary antibodies were purchased from Beijing Bioss Biotechnology Co., Ltd. (Beijing, China) and used at a dilution of 1:1000. The antibodies used for immunohistochemical staining were the same as those used for Western blot analysis.

### 2.8. Mcherry-Lc3b Fluorescence Assay

HT-29 and SW620 cells in the logarithmic growth phase were seeded into 24-well plates at a density of 2 × 10^5^ cells per well and cultured until reaching approximately 70–90% confluence. Cells were transfected with the mCherry-LC3B plasmid using TransIntro EL transfection reagent (TransGen Biotech Co., Ltd. Beijing, China). Briefly, 0.8 μg of mCherry-LC3B plasmid DNA was diluted in 125 μL of serum-free medium, and 1.6 μL of TransIntro EL reagent was added. The mixture was gently mixed and incubated for 20 min at room temperature to allow formation of the plasmid–reagent complex. The complex was then added to the cells, and cells were cultured in a 37 °C, 5% CO_2_ incubator. After 24 h of transfection, cells were treated with DGAE-1 fraction for 48 h. The mCherry-LC3B fluorescence signal was observed and captured using an inverted fluorescence microscope (Nikon, Tokyo, Japan) under identical imaging conditions.

### 2.9. Acridine Orange (Ao) Staining Assay

HT-29 and SW620 cells were seeded into 24-well plates and cultured for 24 h before treatment. Cells were exposed to DGAE-1 fraction alone or in combination with chloroquine (CQ, 10 μM) or 740Y-P (10 μg/mL) for 48 h. After treatment, cells were washed with PBS and stained with an acridine orange (AO) staining kit (Solarbio, Beijing, China) at a final concentration of 1 μg/mL for 15 min at room temperature in the dark. The excess staining solution was removed, and cells were washed with PBS before fluorescence observation. The AO fluorescence signals were captured using an inverted fluorescence microscope (Nikon, Tokyo, Japan) under identical imaging conditions.

### 2.10. Establishment of Mouse Xenotransplantation Model

A total of 24 BALB/C male nude mice, aged 4 weeks and weighing about 20 g (Jiangsu GemPharmatech Biotechnology Co., LTD. Nanjing, China), were raised in the SPF animal culture room of Shanxi Provincial People’s Hospital, with the temperature controlled at 24 ± 1 °C and humidity controlled at 60 ± 5%.

Each nude mouse was injected subcutaneously with 3×10^6^ HT-29 cells suspended in PBS buffer. 7 days after injection, nude mice were randomly divided into three groups (*n* = 8 per group): control group, DGAE-1 fraction treatment group, and 5-fluorouracil (5-FU) group. The sample size was determined based on our previous experience with the same xenograft model and comparable published studies, which indicated that eight mice per group were sufficient to detect biologically meaningful differences in tumor growth. DGAE-1 fraction and 5-FU were freshly prepared in sterile normal saline at a concentration of 2.5 mg/mL before each administration. The prepared solutions remained clear and homogeneous during the administration period without obvious precipitation or phase separation. The DGAE-1 fraction group received DGAE-1 (25 mg/kg) by oral gavage, the 5-FU group received 5-FU (25 mg/kg) by intraperitoneal injection, and the control group received an equal volume of sterile normal saline by oral gavage. All treatments were administered at a dosing volume of 200 μL per mouse every 2 days for 2 weeks.

Tumor size was measured every three days using vernier calipers, and body weight was recorded simultaneously. Tumor volume was calculated using the formula: volume = length × width^2^ × 0.5. After 2 weeks of treatment, all mice were euthanized according to the experimental protocol, and the tumors as well as the heart, liver, spleen, lung, and kidney were collected for subsequent analyses. Humane endpoints were predefined as a tumor volume exceeding 1500 mm^3^ or the appearance of signs of severe distress, and no animals reached these endpoints during the study. All the collected samples were weighed and stored at −80 °C for subsequent investigations.

Animals with unsuccessful tumor establishment or accidental death unrelated to the experimental treatment were predefined for exclusion. No animals met these criteria during the study. All animal experiments carried out in this study strictly adhered to the ethical guidelines and protocols set forth by the Experimental Animal Management and Use Committee of Shanxi University.

### 2.11. Histological and Immunohistochemical Analysis

The tumor tissues collected from nude mice were fixed in 4% paraformaldehyde, dehydrated, embedded in paraffin, and sectioned. The paraffin sections were deparaffinized and rehydrated, followed by antigen retrieval using citrate buffer. After blocking with 3% bovine serum albumin (BSA), the sections were incubated with primary antibodies overnight at 4 °C. The primary antibodies used for immunohistochemical staining were the same as those used for Western blot analysis, with dilution ratios of 1:200. The corresponding antibody information is provided in the Western blot section. After incubation with HRP-conjugated secondary antibodies (1:500) for 1 h at room temperature, the sections were washed and visualized using a diaminobenzidine (DAB) chromogenic detection system. The nuclei were counterstained with hematoxylin, followed by dehydration, mounting, and microscopic examination. Images were captured under identical imaging conditions using a microscope (Servicebio, Wuhan, China). The positive staining area was quantified using ImageJ software. At least five randomly selected fields from each tissue section were analyzed, and the average positive staining area was calculated.

### 2.12. Statistical Analysis

Statistical analysis was performed using GraphPad Prism 8.0 software. Data were presented as mean ± standard deviation (SD). IC_50_ values were calculated using nonlinear regression analysis. Comparisons among multiple groups, including cell viability assays, colony formation assays, apoptosis-related analyses, and Western blot quantification, were performed using one-way ANOVA followed by Tukey’s multiple comparisons test. Tumor growth curves were analyzed using two-way ANOVA. *p* < 0.05 was considered statistically significant. Cell-based experiments were performed with at least three independent replicates. * *p* < 0.05, ** *p* < 0.01.

## 3. Results

### 3.1. Dgae Exhibits Significant Anti-Colorectal Cancer Activity

**Figure 1:**
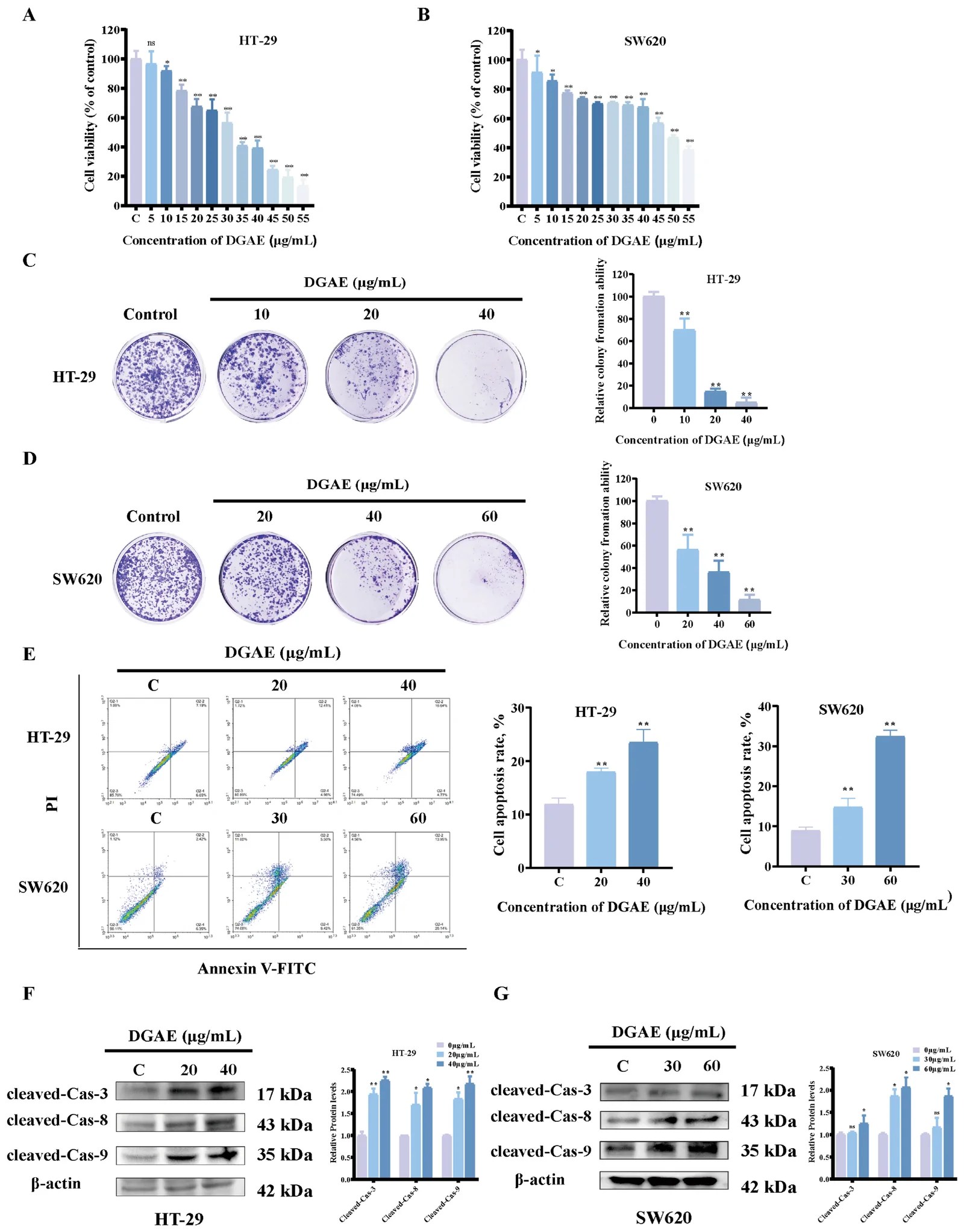
DGAE exhibits significant anti-colorectal cancer activity. (**A**,**B**) HT-29 (**A**) and SW620 (**B**) cells were treated with different concentrations of DGAE, and cell viability was determined by MTT assay after 48 h. (**C**,**D**) HT-29 (**C**) and SW620 (**D**) cells were treated with different concentrations of DGAE for one week, and cell colony formation ability was observed by crystal violet staining. (**E**) HT-29 and SW620 cells were treated with different concentrations of DGAE for 48 h, and the apoptosis rate was determined by Annexin V-FITC/PI double staining followed by flow cytometric analysis. (**F**,**G**) HT-29 (**F**) and SW620 (**G**) cells were treated with different concentrations of DGAE for 48 h. The expression levels of Cleaved-Caspase-3, Cleaved-Caspase-8, Cleaved-Caspase-8 were detected by Western blot, and the relative protein expression was quantified using ImageJ software. Data are presented as mean ± SD from at least three independent experiments. Statistical analysis was performed using one-way ANOVA. * *p* < 0.05, ** *p* < 0.01, and ns indicate no significant difference compared with the control group.

Fenjiu distiller’s grain powder was mixed with 60% ethanol at a solid–liquid ratio of 1:25 and extracted with ultrasonic assistance. After centrifugation and rotary evaporation, the ethanol extract of distiller’s grain was obtained and named DGAE. MTT assay revealed that treatment of colon cancer cells with different concentrations of DGAE for 48 h significantly inhibited cell growth in a dose-dependent manner (Figure 1A,B). The IC_50_ values of DGAE against HT-29 and SW620 cells were 32.820 µg/mL and 48.795 µg/mL, respectively. Colony formation assay further confirmed the inhibitory effect of DGAE on colorectal cells (Figure 1C,D). To explore whether DGAE inhibits colorectal cancer cell proliferation via inducing apoptosis, cells were treated with various concentrations of DGAE for 48 h and subjected to Annexin V-FITC/PI staining. The results showed that the apoptosis rates of both cell lines were significantly increased after DGAE treatment (Figure 1E). Western blot results suggested that the expression levels of cleaved caspase-3, caspase-8 and caspase-9 increased with increasing DGAE concentration (Figure 1F,G), indicating that the caspase-dependent apoptotic pathway was activated in colorectal cancer cells after DGAE treatment. Collectively, these results demonstrate that DGAE induces apoptosis in colorectal cancer cells and thereby inhibits cell proliferation.

### 3.2. Dgae-1 Fraction Is Identified as the Active Fraction Derived from Dgae

**Figure 2:**
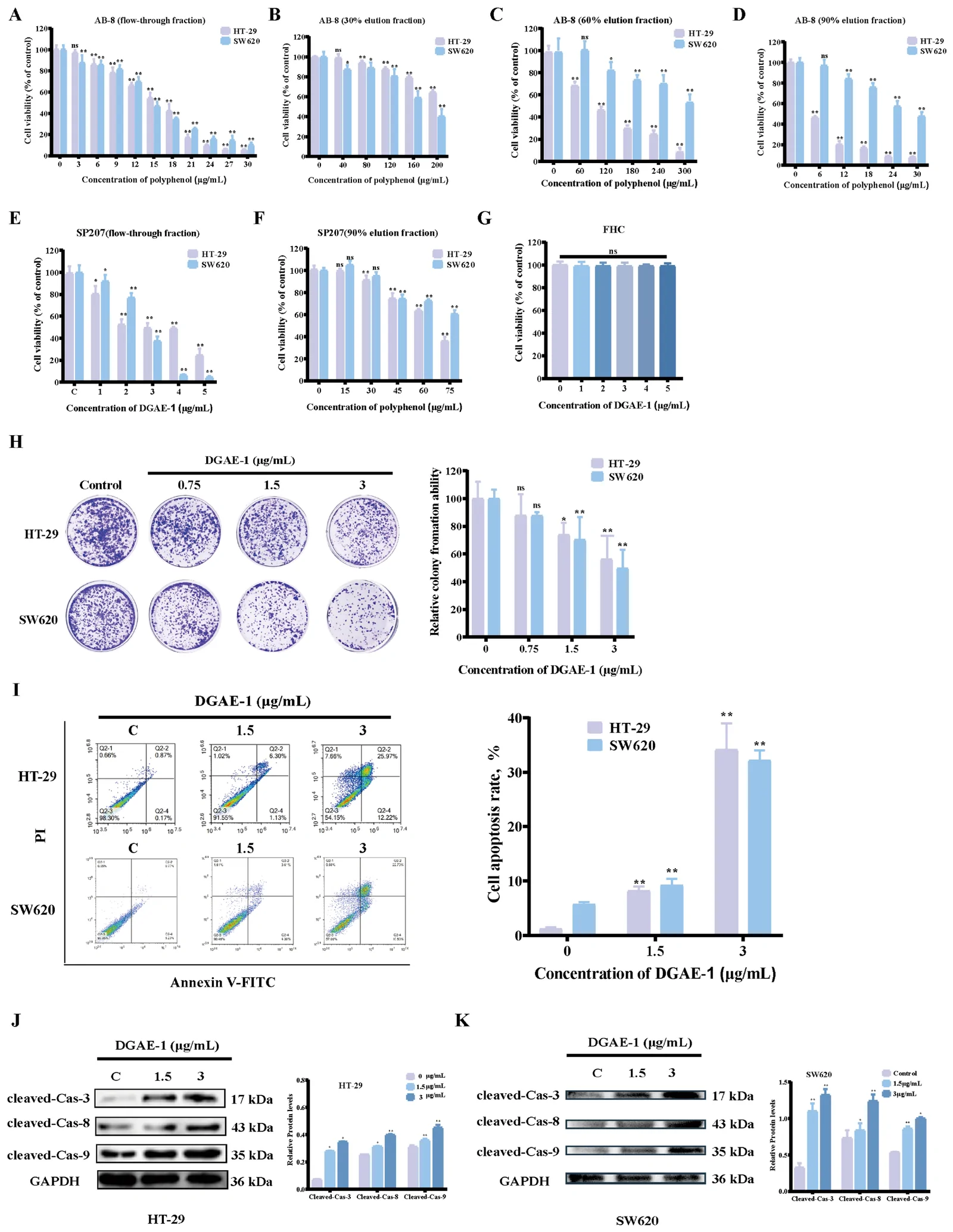
DGAE-1 fraction is identified as the active fraction derived from DGAE. (**A**–**D**) HT-29 and SW620 cells were treated with different fractions eluted from AB-8 macroporous resin for 48 h, and cell viability was determined by MTT assay. (**A**–**D**) represent the permeable fraction, 30%, 60%, and 90% ethanol elution fractions, respectively. (**E**) HT-29 and SW620 cells were treated with the permeable fraction from SP207 macroporous resin for 48 h, and cell viability was determined by MTT assay. (**F**) HT-29 and SW620 cells were treated with the 90% ethanol elution fraction from SP207 macroporous resin for 48 h, and cell viability was determined by MTT assay. (**G**) FHC cells were treated with different concentrations of DGAE-1 fraction for 48 h, and cell viability was determined by MTT assay. (**H**) HT-29 and SW620 cells were treated with different concentrations of DGAE-1 fraction for 7 days, and colony formation ability was evaluated by crystal violet staining. (**I**) HT-29 and SW620 cells were treated with different concentrations of DGAE-1 fraction for 48 h, and apoptosis was analyzed by Annexin V-FITC/PI double staining followed by flow cytometric analysis. (**J**,**K**) HT-29 (**J**) and SW620 (**K**) cells were treated with different concentrations of DGAE-1 fraction for 48 h. The expression levels of cleaved caspase-3, cleaved caspase-8, and cleaved caspase-9 were detected by Western blot analysis. Data are presented as mean ± SD from at least three independent experiments. Statistical analysis was performed using one-way ANOVA. * *p* < 0.05, ** *p* < 0.01, and ns indicate no significant difference compared with the control group.

Macroporous resins possess dual functions of molecular adsorption and molecular sieving, enabling the separation of different substances based on differences in molecular polarity and size. In this study, DGAE was further fractionated using macroporous resins with different polarities to identify the fraction responsible for its anti-colorectal cancer activity. DGAE was loaded onto weakly polar AB-8 macroporous resin and eluted with ultrapure water and gradient ethanol solutions. All fractions were concentrated and evaluated for anti-colorectal cancer activity by MTT assay, and the flow-through fraction showed favorable activity (Figure 2A–D). The flow-through fraction was further separated using nonpolar SP207 macroporous resin. Both the flow-through fraction and the 90% ethanol elution fraction inhibited HT-29 and SW620 cell proliferation, while the flow-through fraction showed lower IC_50_ values (2.673 µg/mL and 2.968 µg/mL) (Figure 2E,F). Based on its stronger anti-colorectal cancer activity, the flow-through fraction was collected, vacuum freeze-dried, and designated as DGAE-1. In addition, DGAE-1 fraction exhibited low cytotoxicity toward normal colonic epithelial FHC cells, as cell viability remained largely unaffected even at the highest tested concentration (5 μg/mL). (Figure 2G). Colony formation assay also demonstrated that DGAE-1 fraction significantly inhibited the proliferation ability of HT-29 and SW620 cells (Figure 2H). Flow cytometric analysis further revealed that DGAE-1 fraction treatment increased the proportion of apoptotic cells in both HT-29 and SW620 cells compared with the control group (Figure 2I). Consistently, Western blot analysis showed that DGAE-1 fraction markedly increased the levels of cleaved caspase-3, cleaved caspase-8, and cleaved caspase-9, indicating the activation of apoptotic signaling pathways (Figure 2J). Therefore, DGAE-1 fraction was identified as the active fraction associated with the anti-colorectal cancer activity of DGAE. The yield of DGAE-1 fraction was approximately 0.56%, with 0.28 g of DGAE-1 fraction obtained from 50 g of dried distillers’ grains powder.

We further characterized the chemical composition of DGAE-1 fraction using liquid chromatography–mass spectrometry (LC-MS). The LC-MS analysis revealed a complex chemical profile of DGAE-1 fraction, and several compounds were putatively annotated based on their MS/MS spectral matches (Table 1). Among the annotated compounds, betaine showed the highest relative peak area (14.578%), followed by 2-amino-1,3,4-octadecanetriol (10.681%). The annotated compounds mainly belonged to carboxylic acids and their derivatives, organic nitrogen compounds, naphthalene derivatives, and other chemical classes. Since DGAE-1 fraction represents a complex mixture and authentic standards were unavailable for most annotated compounds, these annotations were considered tentative identifications rather than absolute confirmation or quantification of individual constituents. These results revealed the chemical profile of DGAE-1 fraction and provided information for further investigation of its active constituents.

### 3.3. Dgae-1 Fraction Induces Autophagy in Colorectal Cancer Cells

**Figure 3:**
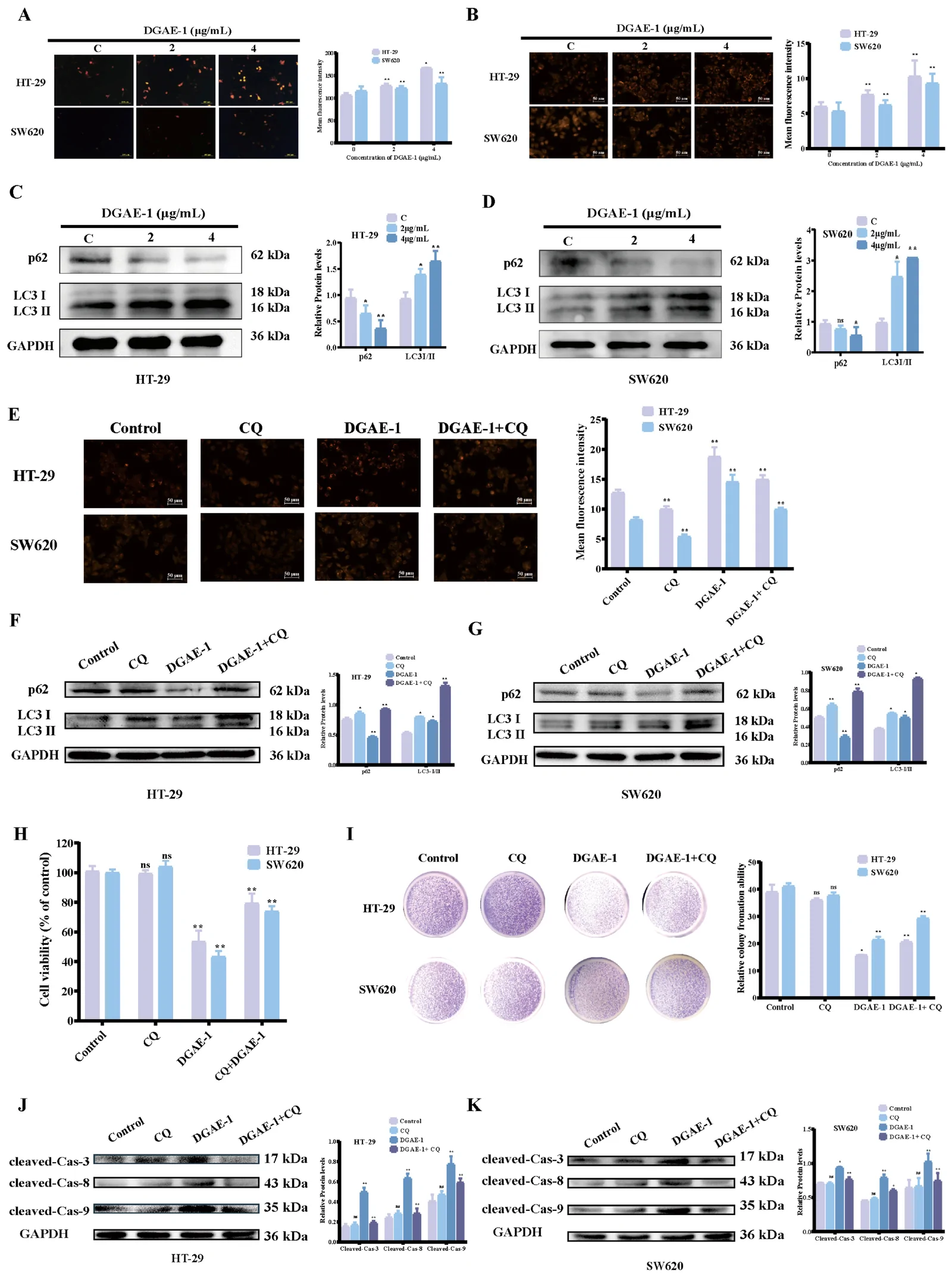
DGAE-1 fraction induces autophagy in colorectal cancer cells. (**A**) HT-29 and SW620 cells were treated with different concentrations of DGAE-1 fraction, and autophagy was evaluated by mCherry-LC3B plasmid transfection assay. Scale bar: 100 μm. (**B**) HT-29 and SW620 cells were treated with different concentrations of DGAE-1 fraction for 48 h, and autophagic vacuoles were detected by acridine orange (AO) staining. Scale bar: 50 μm. (**C**,**D**) HT-29 (**C**) and SW620 (**D**) cells were treated with different concentrations of DGAE-1 fraction for 48 h. The expression levels of LC3 and p62 were detected by Western blot analysis, and relative protein expression was quantified using ImageJ software. (**E**) HT-29 and SW620 cells were treated with DGAE-1 fraction and chloroquine (CQ), and autophagic vacuoles were evaluated by AO staining. Scale bar: 50 μm. (**F**,**G**) HT-29 (**F**) and SW620 (**G**) cells were treated with DGAE-1 fraction, CQ, or their combination for 48 h. The expression levels of LC3 and p62 were detected by Western blot analysis. (**H**) HT-29 and SW620 cells were treated with DGAE-1 fraction, CQ, or their combination for 48 h, and cell viability was determined by MTT assay. (**I**) HT-29 and SW620 cells were treated with DGAE-1 fraction, CQ, or their combination for 7 days, and colony formation ability was evaluated by crystal violet staining. (**J**,**K**) HT-29 (**J**) and SW620 (**K**) cells were treated with DGAE-1 fraction, CQ, or their combination for 48 h. The expression levels of cleaved caspase-3, cleaved caspase-8, and cleaved caspase-9 were detected by Western blot analysis. Data are presented as mean ± SD from at least three independent experiments. Statistical analysis was performed using one-way ANOVA. * *p* < 0.05, ** *p* < 0.01, and ns indicate no significant difference compared with the control group.

Apoptosis and autophagy are distinct but interconnected processes that regulate cell fate. Apoptosis is a major form of programmed cell death, whereas autophagy generally contributes to the maintenance of cellular homeostasis. However, excessive or dysregulated autophagy may interact with apoptotic signaling pathways and contribute to cancer cell death. Therefore, we investigated whether autophagy is involved in the inhibitory effects of DGAE-1 fraction on HT-29 and SW620 cells. Cells were transfected with the mCherry-LC3B plasmid to evaluate changes in autophagy-associated fluorescence signals. As shown in Figure 3A, DGAE-1 fraction treatment resulted in a concentration-dependent increase in mCherry-LC3B fluorescence intensity compared with the control group, suggesting that DGAE-1 fraction promoted autophagy-related changes in colorectal cancer cells. AO staining was further performed to evaluate changes in acidic vesicular organelles. Consistent with the mCherry-LC3B results, AO fluorescence intensity gradually increased following DGAE-1 fraction treatment in a dose-dependent manner (Figure 3B), indicating an increase in acidic vesicular organelles and supporting the enhancement of autophagic activity induced by DGAE-1 fraction. Furthermore, the changes in autophagy-related proteins were examined by Western blotting. After treatment with different concentrations of DGAE-1 fraction for 48 h, the level of LC3-I/II was increased, whereas p62 expression was decreased in colorectal cancer cells (Figure 3C,D), further supporting the induction of autophagic activity by DGAE-1 fraction. To further determine whether autophagy contributes to the anti-colorectal cancer effects of DGAE-1 fraction, chloroquine (CQ) was used to inhibit autophagic degradation. As shown in Figure 3E, AO staining showed that CQ treatment altered the AO fluorescence changes induced by DGAE-1 fraction, suggesting that CQ interfered with the autophagic process regulated by DGAE-1 fraction. Western blot analysis further revealed that, compared with DGAE-1 fraction treatment alone, co-treatment with CQ resulted in increased LC3-I/II accumulation and elevated p62 expression (Figure 3F,G), indicating that CQ inhibited autophagic degradation and affected autophagic flux. We next investigated whether autophagy inhibition affected the biological effects of DGAE-1 fraction. Compared with DGAE-1 fraction treatment alone, CQ co-treatment partially restored cell viability (Figure 3H), suggesting that inhibition of autophagy weakened the cytotoxic effect of DGAE-1 fraction. Consistently, colony formation assays showed that CQ treatment partially reversed the inhibitory effect of DGAE-1 fraction on colony-forming ability (Figure 3I), indicating that autophagy contributes to the suppression of colorectal cancer cell proliferation by DGAE-1 fraction. Furthermore, the expression levels of apoptosis-related proteins were analyzed. Compared with the DGAE-1 fraction group, the DGAE-1/CQ co-treatment group showed reduced levels of cleaved caspase-3, cleaved caspase-8, and cleaved caspase-9 (Figure 3J,K), suggesting that inhibition of autophagy attenuated DGAE-1 fraction-induced apoptotic responses. Collectively, these results were consistent with the expected effects of autophagy inhibition and suggested that autophagy activation contributes to the anti-colorectal cancer effects of DGAE-1 fraction.

### 3.4. DGAE-1 Fraction Regulates Autophagy Through the PI3K/AKT Signaling Pathway in Colorectal Cancer Cells

**Figure 4:**
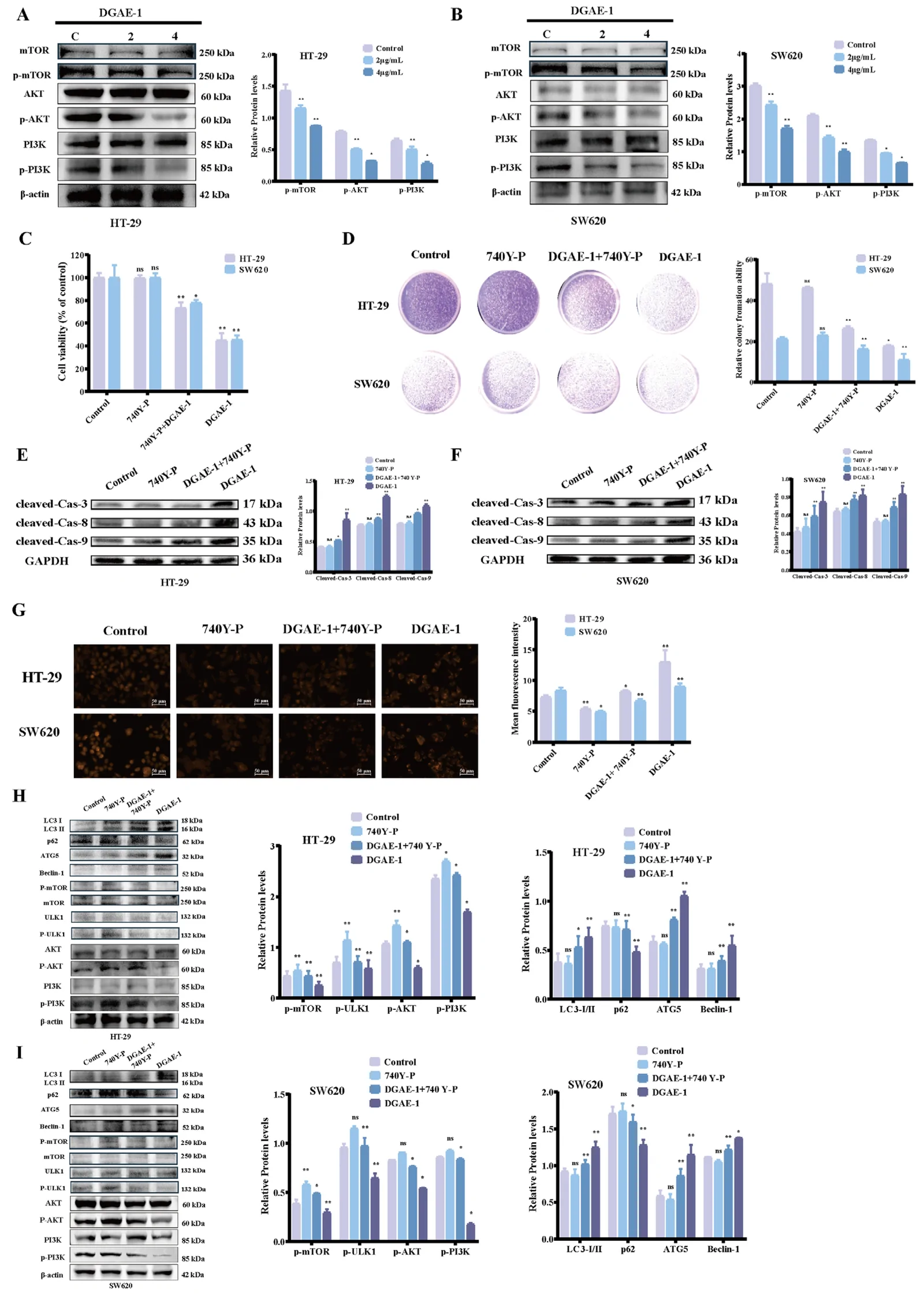
DGAE-1 fraction regulates autophagy through the PI3K/AKT signaling pathway in colorectal cancer cells. (**A**,**B**) HT-29 (**A**) and SW620 (**B**) cells were treated with different concentrations of DGAE-1 fraction for 48 h. The expression levels of phosphorylated and total PI3K, AKT, and mTOR were detected by Western blot analysis. Phosphorylated protein levels were normalized to their corresponding total protein levels, and relative protein expression was quantified. (**C**) HT-29 and SW620 cells were treated with DGAE-1 fraction, 740Y-P, or their combination for 48 h, and cell viability was determined by MTT assay. (**D**) HT-29 and SW620 cells were treated with DGAE-1 fraction, 740Y-P, or their combination for 7 days, and colony formation ability was evaluated by crystal violet staining. (**E**,**F**) HT-29 (**E**) and SW620 (**F**) cells were treated with DGAE-1 fraction, 740Y-P, or their combination for 48 h. The expression levels of cleaved caspase-3, cleaved caspase-8, and cleaved caspase-9 were detected by Western blot analysis. (**G**) HT-29 and SW620 cells were treated with DGAE-1 fraction, 740Y-P, or their combination for 48 h, and autophagy-related changes were evaluated by acridine orange (AO) staining. Scale bar: 50 μm. (**H**,**I**) HT-29 (**H**) and SW620 (**I**) cells were treated with DGAE-1 fraction, 740Y-P, or their combination for 48 h. The expression levels of phosphorylated and total PI3K, AKT, mTOR, and ULK1, as well as autophagy-related proteins LC3, p62, Beclin-1, and ATG5, were detected by Western blot analysis. Phosphorylated protein levels were normalized to their corresponding total protein levels, while autophagy-related protein levels were normalized to the internal control. Relative protein expression was quantified using ImageJ software. Data are presented as mean ± SD from at least three independent experiments. Statistical analysis was performed using one-way ANOVA. * *p* < 0.05, ** *p* < 0.01, and ns indicate no significant difference compared with the control group.

The PI3K/AKT/mTOR signaling pathway is an important regulator of autophagy, and inhibition of mTOR activity is closely associated with autophagy induction. We next examined whether this signaling pathway was involved in the autophagy regulation by DGAE-1 fraction. Western blot analysis revealed that DGAE-1 fraction treatment markedly decreased the phosphorylation levels of PI3K, AKT, and mTOR, whereas the total protein levels of PI3K, AKT, and mTOR remained largely unchanged (Figure 4A,B). These results indicated that DGAE-1 fraction suppressed the activation of the PI3K/AKT/mTOR signaling pathway in colorectal cancer cells. To determine whether PI3K/AKT signaling contributes to the biological effects of DGAE-1 fraction, 740Y-P was used as a PI3K activator. Compared with DGAE-1 fraction treatment alone, 740Y-P co-treatment partially restored cell viability and colony formation ability (Figure 4C,D), indicating that activation of PI3K/AKT signaling partially rescued the inhibitory effects of DGAE-1 fraction on colorectal cancer cell growth. Consistently, the levels of cleaved caspase-3, cleaved caspase-8, and cleaved caspase-9 were reduced after 740Y-P co-treatment compared with the DGAE-1 fraction group (Figure 4E,F), suggesting that PI3K/AKT activation partially rescued DGAE-1 fraction-induced apoptotic changes. Since DGAE-1 fraction-mediated autophagy contributes to its anti-colorectal cancer effects, we further investigated whether PI3K/AKT activation affected the autophagic response induced by DGAE-1 fraction. As shown in Figure 4G, 740Y-P treatment reduced the increase in AO fluorescence intensity caused by DGAE-1 fraction, suggesting that activation of PI3K/AKT signaling partially reversed the autophagy-associated changes induced by DGAE-1 fraction. Western blot analysis was further conducted to evaluate the changes in the PI3K/AKT/mTOR/ULK1 signaling axis and autophagy-related proteins. Compared with DGAE-1 fraction treatment alone, 740Y-P co-treatment restored the phosphorylation levels of PI3K, AKT, and mTOR, accompanied by increased ULK1 Ser757 phosphorylation (Figure 4H,I), suggesting that activation of PI3K/AKT/mTOR signaling affected the autophagic response regulated by DGAE-1 fraction. Consistently, 740Y-P treatment partially reversed the alterations in autophagy-related proteins, as evidenced by reduced LC3-I/II accumulation and Beclin-1/ATG5 expression, together with increased p62 levels (Figure 4H,I). These results further support the involvement of the PI3K/AKT/mTOR/ULK1 signaling axis in mediating the autophagic changes induced by DGAE-1 fraction.

### 3.5. DGAE-1 Fraction Suppresses Colorectal Cancer Xenograft Growth in Nude Mice

**Figure 5:**
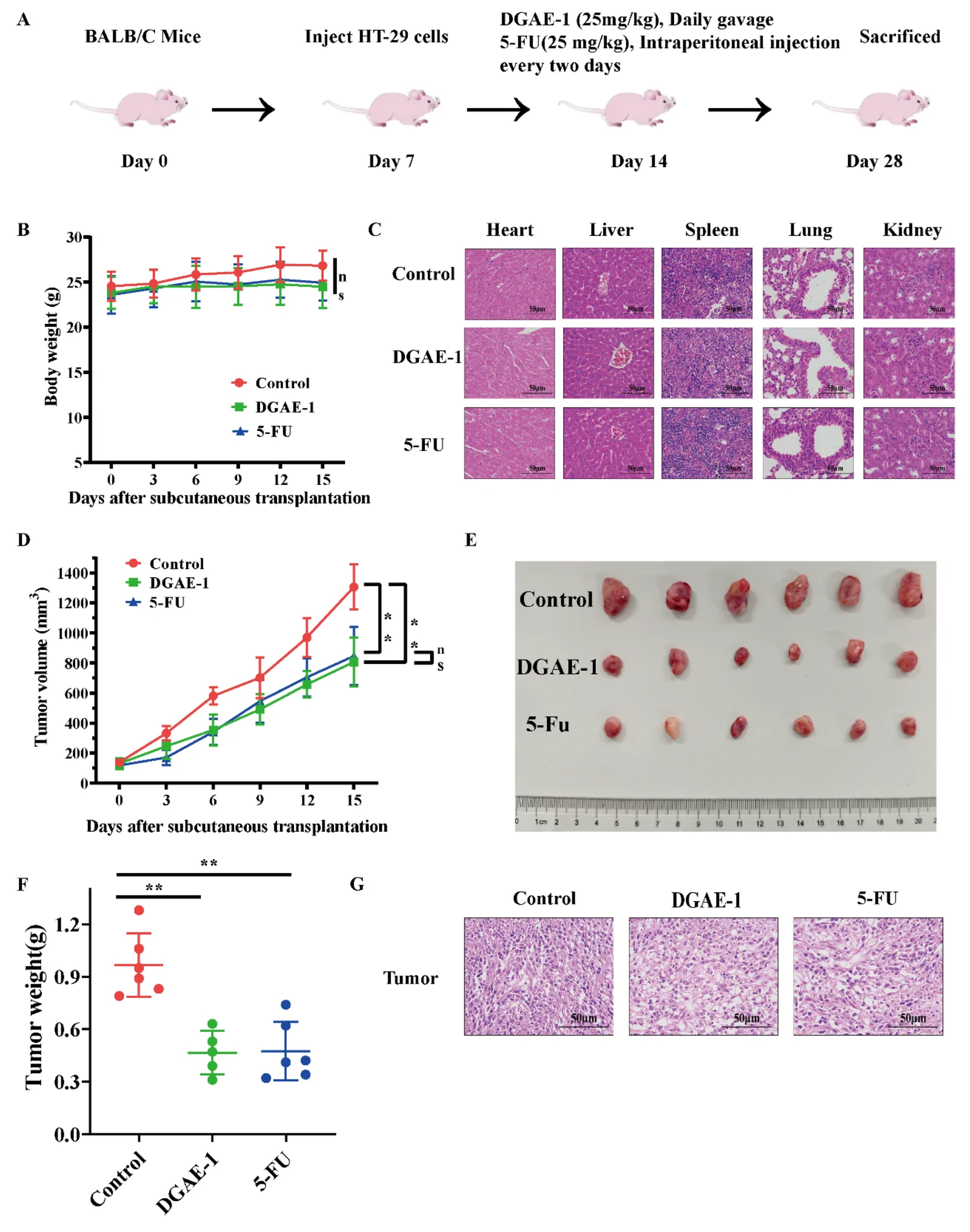
DGAE-1 fraction suppresses colorectal cancer xenograft growth in nude mice. (**A**) Overall design of the animal experiments. (**B**) The body weight of nude mice in each group was recorded. ns means not significant. (**C**) H&E staining was used to evaluate the effects of DGAE-1 treatment on major organs in nude mice. H&E staining, 400× magnification; scale bar: 50 µm. (**D**) The tumor growth curve in nude mice was plotted according to tumor volume. ** *p* < 0.01, ns means not significant. (**E**) Representative images of xenograft tumors in each group. (**F**) Measurement of tumor weight from nude mice. (**G**) H&E staining was performed to observe the effect of DGAE-1 treatment on the morphology of tumor tissues in nude mice. H&E staining, 400× magnification; scale bar: 50 µm. Data are presented as mean ± SD from at least three independent experiments. Statistical analysis was performed using one-way ANOVA. ** *p* < 0.01, and ns indicate no significant difference compared with the control group.

To further evaluate the in vivo anti-colorectal cancer activity of DGAE-1 fraction, a nude mouse xenograft model was established using human colorectal cancer HT-29 cells. A 5-FU treatment group was included as a positive control, and DGAE-1 fraction was administered by intragastric gavage (Figure 5A). During the treatment period, no significant changes in body weight or abnormal behaviors were observed in mice (Figure 5B). H&E staining of major organs, including the heart, liver, spleen, lung, and kidney, showed no obvious pathological alterations after treatment, suggesting that DGAE-1 fraction did not induce apparent histopathological damage under the tested conditions (Figure 5C). Tumor growth was monitored throughout the administration period, and the corresponding tumor growth curves were generated. Compared with the control group, DGAE-1 fraction treatment significantly inhibited tumor growth, as indicated by the reduced tumor volume and tumor weight (Figure 5D–F). Furthermore, H&E staining of tumor tissues showed that tumors in the control group exhibited densely packed cellular structures, whereas DGAE-1 fraction-treated tumors displayed relatively loose tissue organization (Figure 5G). Collectively, these results demonstrate that DGAE-1 fraction effectively suppresses colorectal cancer xenograft growth in vivo.

### 3.6. Dgae-1 Fraction Regulates Autophagy Through the Pi3k/Akt Signaling Pathway In Vivo

**Figure 6:**
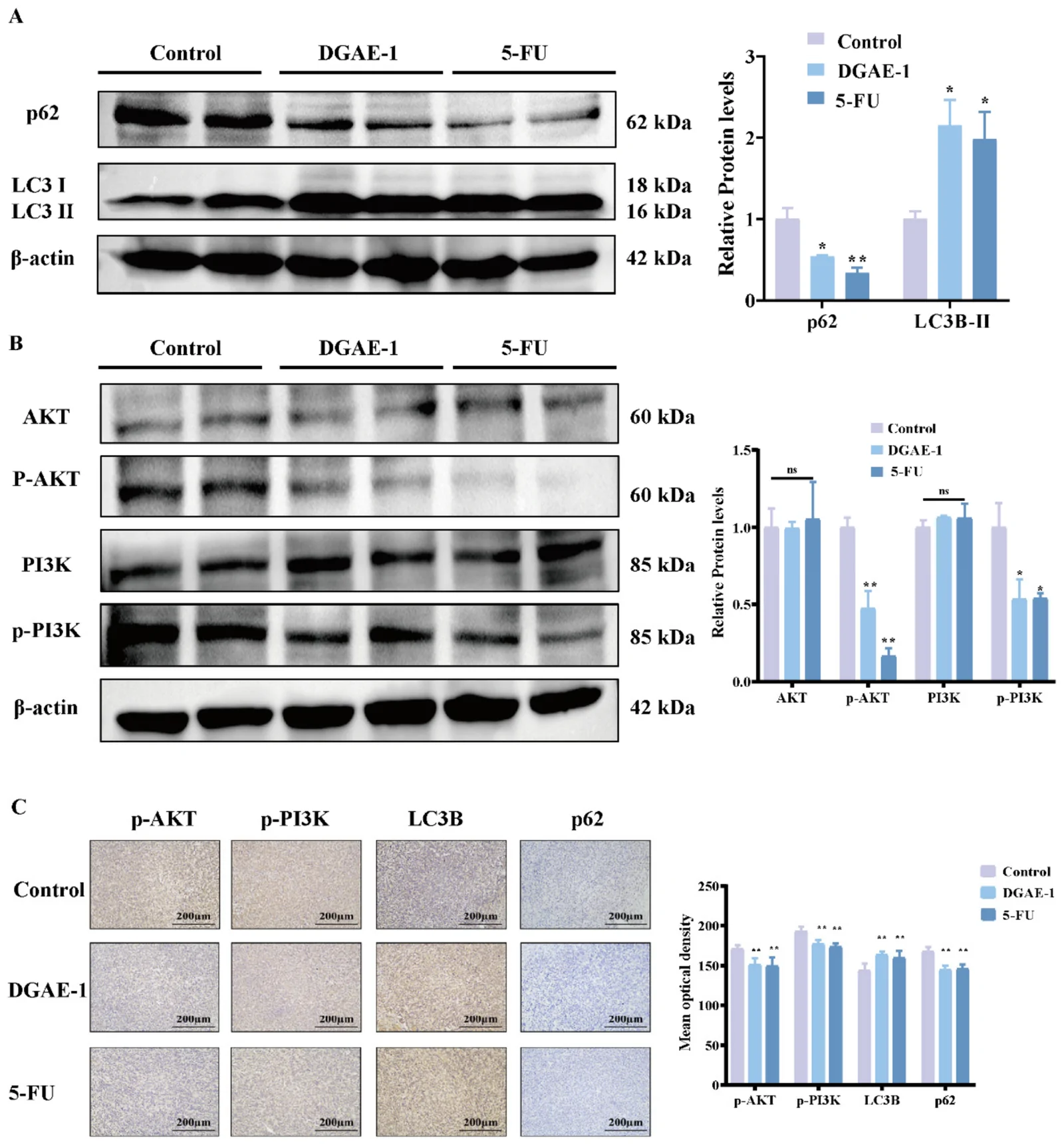
DGAE-1 fraction regulates autophagy through the PI3K/AKT signaling pathway in vivo. (**A**,**B**) The expression of LC3B, p62, phosphorylated and total PI3K/AKT in tumor tissues of DGAE-1-treated nude mice was detected by Western blot. (**C**) The expression levels of phosphorylated PI3K, AKT, LC3B and p62 in tumor tissues were detected by immunohistochemical staining. IHC 100×, scale: 200 µm. Data are presented as mean ± SD from at least three independent experiments. Statistical analysis was performed using one-way ANOVA. * *p* < 0.05, ** *p* < 0.01, and ns indicate no significant difference compared with the control group.

To further investigate the involvement of autophagy and PI3K/AKT signaling in the antitumor effect of DGAE-1 fraction in vivo, Western blot analysis was performed to examine the levels of autophagy-related proteins and PI3K/AKT pathway markers in xenograft tumor tissues. Compared with the control group, DGAE-1 fraction treatment significantly decreased the levels of p62, p-AKT, and p-PI3K, accompanied by an increase in LC3B-II levels (Figure 6A,B). Immunohistochemical staining was further performed to detect the expression of LC3B, p62, p-AKT, and p-PI3K in xenograft tissues. The IHC results showed similar expression trends to those observed in Western blot analysis (Figure 6C,D). These findings indicate that DGAE-1 fraction induces autophagy and inhibits PI3K/AKT signaling in colorectal cancer xenografts, consistent with the observations obtained in vitro.

## 4. Discussion

Colorectal cancer (CRC) remains a major therapeutic challenge [20]. Food-processing by-products are increasingly recognized as promising sources of bioactive compounds, offering new opportunities for the discovery of functional ingredients and their value-added utilization [21,22]. Distillers’ grains, one of the major by-products generated during the brewing process, have traditionally been used for animal feed [23], fertilizers [24], and bioenergy production [25]. However, increasing evidence suggests that distillers’ grains contain diverse bioactive constituents with antioxidant, anti-inflammatory, immunomodulatory, and metabolic activities [8]. Despite these findings, their potential anti-tumor properties, particularly against colorectal cancer, remain insufficiently explored. In the present study, we obtained a bioactive fraction (DGAE-1 fraction) from Fenjiu distillers’ grains and demonstrated its inhibitory effects on colorectal cancer cell growth and xenograft tumor growth. These findings expand the understanding of the potential value of Fenjiu distillers’ grains as a source of bioactive fractions and provide a basis for further investigation of their functional properties.

Autophagy is a highly conserved intracellular degradation process that plays a complex and context-dependent role in cancer progression. Although autophagy can support tumor cell survival under metabolic stress, excessive or sustained autophagic activation may contribute to cellular damage and tumor suppression [26]. Therefore, modulation of autophagy has emerged as an important strategy for exploring new anticancer mechanisms of natural products [27,28,29]. In the present study, the observed changes in autophagy-related markers and fluorescence signals following DGAE-1 fraction treatment, together with the effects of chloroquine-mediated autophagy inhibition, suggest that autophagy may contribute to the anti-colorectal cancer activity of DGAE-1 fraction. However, whether autophagy induction represents the primary mechanism responsible for cell death or functions as a contributing process requires further validation using genetic approaches targeting key autophagy-related genes.

The PI3K/AKT signaling pathway is frequently dysregulated in colorectal cancer and plays an important role in regulating cell proliferation, survival, and autophagy [30]. Within this signaling network, the mTOR-ULK1 axis is considered an important regulator of autophagy initiation, and modulation of this axis has been reported to influence autophagic responses in cancer cells [31]. In this study, DGAE-1 fraction was found to regulate the PI3K/AKT/mTOR/ULK1 signaling axis and modulate autophagy-related responses in colorectal cancer cells. Furthermore, activation of PI3K/AKT signaling by 740Y-P partially attenuated the biological effects induced by DGAE-1 fraction, including the changes in autophagy-related responses. These findings suggest that the PI3K/AKT/mTOR/ULK1 signaling axis is involved in DGAE-1 fraction-induced autophagy regulation. Nevertheless, the current data do not establish this signaling axis as the sole mediator of the observed effects, and further genetic studies are required to clarify the detailed molecular mechanisms.

Despite the biological effects observed in this study, several limitations should be considered. First, DGAE-1 fraction represents a complex mixture containing multiple tentatively annotated constituents, and the specific compounds responsible for its anti-colorectal cancer activity remain unclear. LC-MS analysis provided preliminary information regarding the chemical composition of DGAE-1 fraction. Several classes of compounds were tentatively annotated, including carboxylic acids and derivatives, organic nitrogen compounds, and organic phosphoric acid derivatives. Among the annotated constituents, betaine exhibited a relatively high relative abundance. Betaine has been reported to participate in methyl metabolism, oxidative stress regulation, and inflammatory responses, processes that are closely associated with tumor development [32]. Other annotated metabolites, such as L-isoleucine and L-pyroglutamic acid, have been associated with metabolic regulation and cellular stress responses and may contribute to the overall biological activity of DGAE-1 fraction [33,34]. However, the biological activity of a complex fraction cannot be directly attributed to the compounds with the highest abundance. Components with lower relative abundance may also contribute to the overall activity through synergistic or additive interactions. Therefore, further studies are required to isolate individual constituents, validate their biological activities, and clarify the relationship between chemical composition and the anti-colorectal cancer effects of DGAE-1 fraction. Among the tentatively annotated constituents of DGAE-1 fraction, 1,3,5-trinitroso-1,3,5-triazinane was detected with a relatively high relative abundance. However, considering that the identification was based on MS/MS spectral matching and database annotation, the presence of this compound requires further confirmation using authentic standards and additional analytical approaches. The possible origin of this annotation remains unclear and may be related to complex chemical transformations during fermentation, extraction, or database-based spectral matching. Therefore, its potential contribution to the biological activity of DGAE-1 fraction cannot be determined at this stage. Further studies are required to validate its identity and clarify its biological relevance.

Another limitation is that the pharmacokinetic behavior and biological availability of the active constituents after oral administration remain unknown. In the xenograft model, oral administration of DGAE-1 fraction inhibited tumor growth under the experimental conditions, and no obvious histopathological alterations were observed in major organs. However, the absorption, metabolism, distribution, and tumor tissue exposure of the active constituents have not been determined. This issue is particularly important because several tentatively annotated compounds, including amino acid-related metabolites and betaine, exhibit relatively high polarity, which may influence their bioavailability and tissue distribution. Therefore, pharmacokinetic and tissue-distribution studies are needed to further understand the biological availability and potential applications of DGAE-1 fraction.

Overall, DGAE-1 fraction should be considered a complex bioactive fraction rather than a single purified compound. Therefore, the biological effects observed in this study likely reflect the combined activities of multiple constituents within this fraction. Although DGAE-1 fraction showed biological activity against colorectal cancer in cellular and xenograft models, further studies using additional disease models, comprehensive safety evaluations, pharmacokinetic analyses, and molecular target validation are required to better understand its biological properties. These investigations will help identify the active constituents, clarify the underlying mechanisms, and further clarify the feasibility of utilizing Fenjiu distillers’ grains as a source of bioactive functional ingredients.

## 5. Conclusions

In conclusion, this study demonstrates that DGAE-1, a bioactive fraction derived from Fenjiu distillers’ grains, suppresses colorectal cancer progression by inhibiting the PI3K/AKT signaling pathway and promoting autophagy. These findings suggest that DGAE-1 may represent a potential bioactive resource for colorectal cancer intervention. Since the chemical constituents of DGAE-1 have only been tentatively identified, the key active components responsible for its anti-colorectal cancer effects and autophagy regulation remain to be determined. Future studies should focus on further separation and identification of the major active constituents in DGAE-1, clarifying the contribution of individual components to its overall biological activity, validating their anti-tumor effects, and elucidating the underlying mechanisms and direct targets. These efforts will provide a deeper understanding of the bioactive basis and therapeutic potential of DGAE-1.

## Figures and Tables

**Table 1:** Tentatively identified compounds in DGAE-1 based on LC-MS analysis (positive ion mode).

Class	Name	RT (min)	CAS-Num	Percentage (%)
Carboxylic acids and derivatives	Betaine	0.846	107-43-7	14.578
	L-Isoleucine	1.284	73-32-5	7.276
	L-Pyroglutamic acid	1.127	98-79-3	6.652
	D-(+)-Proline	0.828	344-25-2	3.605
	L-Alanyl-L-proline	1.125	13485-59-1	1.060
	L-Glutamic acid	0.977	56-86-0	1.015
	DL-Lysine	0.717	70-54-2	0.800
	L-Histidine	1.089	71-00-1	0.582
	DL-Stachydrine	0.827	4136-37-2	0.514
	4-Guanidinobutyric acid	1.08	463-00-3	0.506
	Ala-Val	1.183	3303-45-5	0.468
	D-(+)-Pipecolinic acid	0.822	1723-00-8	0.348
	Glycyl-L-proline	1.061	704-15-4	0.222
	N-Alpha-acetyllysine	0.871	1946-82-3	0.193
	Leucylalanine	1.309	67368-02-9	0.115
	Cyclo-prolylglycine	1.39	97011-16-0	0.084
	Isoleucylglutamate	1.229	NA	0.079
	2-Propanamidoacetic acid	1.145	21709-90-0	0.028
Organonitrogen compounds	2-Amino-1,3,4-octadecanetriol	10.303	554-62-1	10.681
	MFCD00059002	10.251	1541-67-9	10.021
	Choline	0.803	62-49-7	3.489
	Lauryldimethylamine oxide	10.29	1643-20-5	0.879
	Hydrolyzed fumonisin B1	10.357	145040-09-1	0.841
	N,N-Diisopropylethylamine (DIPEA)	4.869	7087-68-5	0.060
Naphthalenes	2H-Naphtho[1,8-bc]thiophene	0.795	209-11-0	10.578
Triazinanes	1,3,5-Trinitroso-1,3,5-triazinane	0.794	13980-04-6	10.459
Organic phosphoric acids and derivatives	N-methylethanolamine phosphate	0.781	NA	8.551
Pyridines and derivatives	Methyl isonicotinate	0.817	2459-09-8	1.434
	4-Pyridoxate	1.31	82-82-6	0.055
Quinolines and derivatives	Xanthurenic acid	0.803	59-00-7	1.170
Cinnamic acids and derivatives	2-Hydroxycinnamic acid	1.161	614-60-8	0.055

## Data Availability

The original contributions presented in this study are included in the article/Supplementary Material. Further inquiries can be directed to the corresponding author.

## Supplementary Materials

The following supporting information can be downloaded at: https://www.mdpi.com/article/10.3390/foods15162834/s1, Figure S1: Total ion chromatogram of DGAE-1 acquired under positive electrospray ionization mode (ESI+); Figure S2: Schematic illustration of the preparation and purification of DGAE-1 fraction from Fenjiu distillers’ grains.
