# Supplementary material for: Distillers’ Grains-Derived Bioactive Fraction Suppresses Colorectal Cancer Progression Through Modulation of Autophagy-Associated PI3K/AKT Signaling
Source: Foods. 2026 Aug 14;15(16):2834. doi: 10.3390/foods15162834 (PMC13512201; doi:10.3390/foods15162834)
Supplement: Supplementary file 1 [file foods-15-02834-s001.zip › foods-4431193-supplementary.pdf]

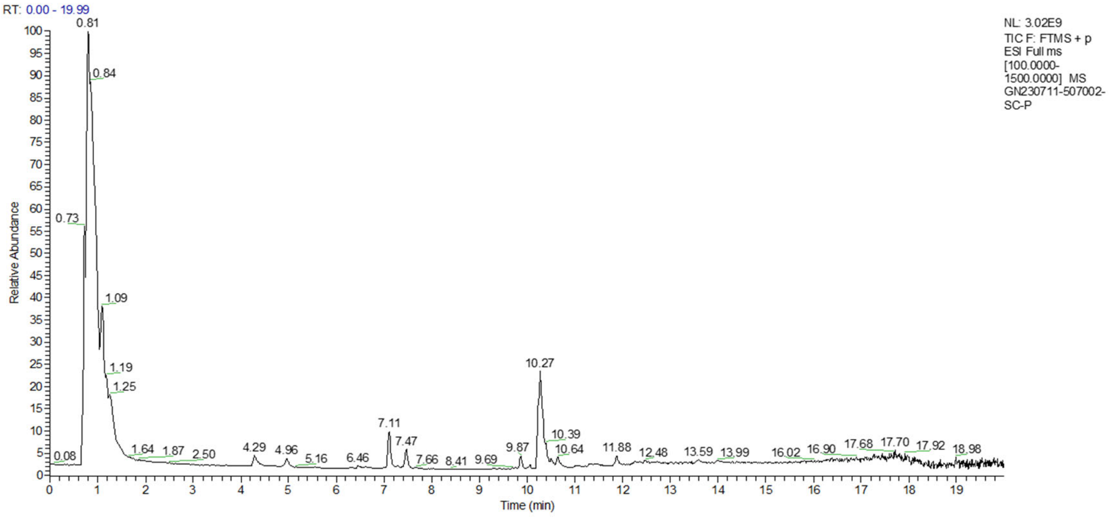

Figure S1. Total ion chromatogram of DGAE-1 acquired under positive electrospray ionization mode (ESI+).

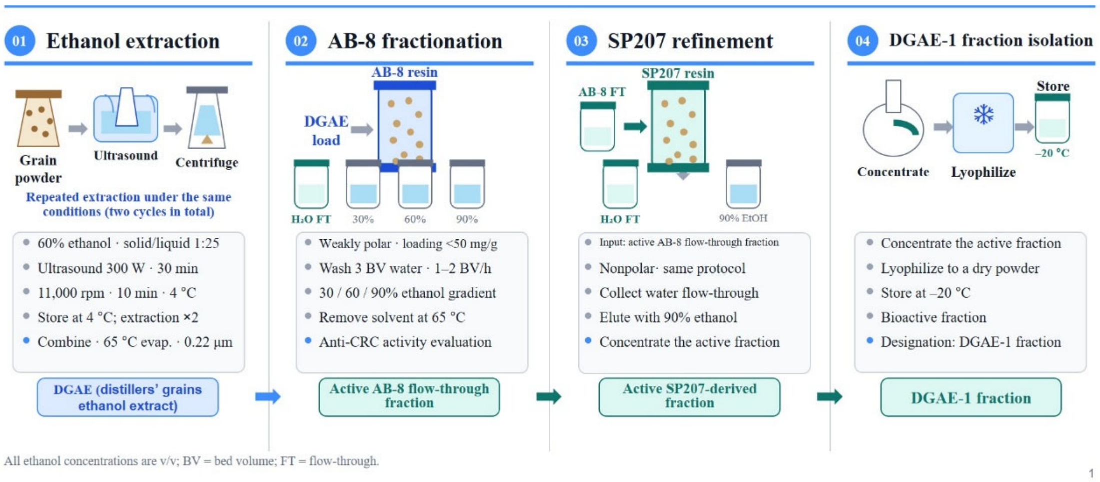

Figure S2. Schematic illustration of the preparation and purification of DGAE-1 fraction from Fenjiu distillers' grains.
